# Assessing the links between human rights and global tobacco control through statements made on global fora

**DOI:** 10.1186/s12889-021-10451-2

**Published:** 2021-03-04

**Authors:** Neiloy R. Sircar, Stella A. Bialous

**Affiliations:** 1 Center for Tobacco Control Research and Education, University of California, San Francisco, 530 Parnassus Ave, San Francisco, CA 94143 USA; 2 Department of Social and Behavioral Sciences, School of Nursing, University of California, San Francisco, 530 Parnassus Ave, San Francisco, CA 94143 USA

**Keywords:** Global health, Public health, Tobacco control, Human rights, International law

## Abstract

**Background:**

Increasingly, international health bodies frame public health measures, including tobacco control, in the context of human rights (HR). It is unclear how prevalent is the connection between human rights and tobacco control within global health governance. This paper describes the inclusion of HR in tobacco control governance, and the inclusion of tobacco control in HR treaty oversight. We depict the current reach of HR’s normative influence in framing the tobacco epidemic in global, regional, and country-specific contexts.

**Methods:**

We reviewed documents (agenda, reports) from 2010 to 2019 from the World Health Assembly (WHA); the WHO Western Pacific Regional Committee Meetings (RCM); the WHO Framework Convention on Tobacco Control (WHO FCTC) Conferences of the Parties (COP); and documents provided by Pacific Island Countries party to, or by committees overseeing, HR treaties. We purposively selected the Western Pacific Region, and Pacific Island Countries specifically, to represent countries of varying populations, capacities, and governance.

**Results:**

Tobacco control and HR are infrequently mentioned together in the WHAs, and primarily in only one COP. Tobacco control is mentioned in 47 HR treaty committee documents for Pacific Island Countries, mostly under the Convention of the Rights of the Child recognizing or calling for ratification of the WHO FCTC. HR and tobacco control are connected in WHO Western Pacific RCM, particularly through their two most-recent action plans adopted by respective RCMs.

**Discussion:**

Tobacco control as a HR concern is gaining traction within HR treaty bodies, at least with respect to children’s health in the Western Pacific Region.

**Conclusion:**

Globally, HR is just emerging as an influence in global health governance for tobacco discussions. Within the Western Pacific Region however tobacco control is seen by some authorities as a HR issue. Similarly, to HR experts, tobacco control is becoming important to how Pacific Island Countries fulfill their treaty obligations, suggesting tobacco control advocates might explore these mechanisms to further influence the development of strong tobacco control measures to implement the WHO FCTC.

## Background

The human right to health and its subsidiary rights are increasingly being promoted as a component of tobacco control policies, and present opportunities for law and public health programs to effectuate healthy behaviors and environments [1, 2]. The WHO Framework Convention on Tobacco Control (WHO FCTC) is a United Nations (UN) treaty that frames its obligations as reinforcing human rights [3]. Further, the Sustainable Development Goals (SDG), including SDG 3: Healthy lives and wellbeing and SDG 3.a.: strengthening implementation of the WHO FCTC, are linked to human rights [4]. The 2015 UN General Assembly explicitly stated that “[T] he new [SDG] Agenda … is grounded in the Universal Declaration of Human Rights, international human rights treaties … [and] is informed by other instruments such as the Declaration on the Right to Development” [5]. Public health law, and its relationship to human rights law, is essential to fulfilling the WHO FCTC, the SDG and creating enabling environments for the realization of human rights for health [6, 7].

UN human rights treaties establish treaty bodies to monitor compliance and implementation of those treaties’ obligations, and oversee treaty growth and continued relevance [8]. In addition, the UN Human Rights Council oversees the Universal Periodic Review (UPR) [9], a report submitted by all countries that are members of the UN, assessing each State’s human rights fulfillment. The UN Human Rights Council maintains reports and documents for public access with respect to the human rights treaty bodies and State fulfillment of a given treaty’s provisions [10].

Human rights are recognized in law and global norms, and the most fundamental of rights are “non-derogable” – meaning they may not be curtailed for anyone at any time [11]. The human right to health is recognized as the core human right to life, and speaks to both an individual’s as well as the public’s health [12]. However, racial and ethnic minorities, low-income groups, women, and children often experience intersectional forms of discrimination and stigma impacting their rights-realization. There are several human rights treaties that specify closing health disparities and promoting health, including the International Covenant on Economic, Social, and Cultural Rights (ICESCR), Convention on the Elimination of All Forms of Discrimination against Women (CEDAW) and Convention on the Rights of the Child (CRC).

The tobacco industry has a history of targeting the above-mentioned groups, who suffer greater tobacco-related harms when compared to the general population [13]. Tobacco control policies reflected in the WHO FCTC are essential tools for addressing disparities as matters of public health and human rights equity. Non-governmental organizations and civil society advocacy groups have been instrumental in linking human rights and disparities in tobacco-related harms in national and international forums [14, 15].

### Tobacco control and human rights

Tobacco control has been described as a matter of human rights and particularly the right to health [16–18]. The connection of human rights to tobacco control measures is attributed to a dedicated yet, as Reubi puts it, “small, international network of public health experts and lawyers” that melded human rights rhetoric with tobacco-related health concerns (e.g. secondhand smoking, protection from predatory marketing, accountability in policymaking) [16]. Applying established international human rights mechanisms, including the Universal Declaration of Human Rights, CEDAW, CRC, and the foundational treatise ICESCR, to tobacco control created new opportunities for public health to advocate for strong tobacco control measures [15–23]. Similarly, human rights law advocates have achieved at least some success in raising the profile of tobacco control in human rights. The World Health Organization (WHO)’s “Advancing the Right to Health: The Vital Role of Law,” published in 2017, notes that human rights treaties – and especially, CRC and CEDAW – can and have been used to promote health, and separately the need for public health law and regulation to effectuate the WHO FCTC [24]. On 27 July 2020, the UN Special Rapporteur for the right to health stated that front-of-packing labelling for tobacco products was an “essential component” of the policies countries are obligated to enact in order to fulfill their duties under human rights law (particularly the CRC) [25]. However, it is unclear if tobacco control policy decisionmakers (the States themselves) consider tobacco control a human rights concern.

The WHO FCTC – though not a human rights treaty – was framed by countries as implementing their human rights commitments [16, 20, 26]. WHO FCTC Articles, including Articles 5.3, 8, 13, and 18, create obligations on governments to protect public health from tobacco product exposure and tobacco industry influences which favor tobacco over the public’s health. Human rights law advocates construe these articles and the WHO FCTC overall as implementation devices for a human rights-based approach to health and have argued that failure to adequately provide effective tobacco control measures is a human rights violation [27]. Even in countries that are not Parties to the WHO FCTC (notably, the United States and its dependencies) civil society groups have used the human rights framing to advocate for stronger tobacco control policies protecting vulnerable populations covered in other treaties – such as women [15]. Thus, WHO FCTC as a global treaty stands to offer significant normative value to human rights [6].

The purpose of this paper is to describe the inclusion – and absences – of human rights and tobacco mentions in global health discussions among State actors. Our specific objectives include 1) describing the frequency of mentions of human rights and tobacco control (and related key words, e.g. smoking), in reference to the other, in World Health Assembly (WHA) meetings from 2010 to 2019; 2) describing the aforementioned frequency of mentions in WHO FCTC Conference of the Parties (COP) from 2010 to 2019; 3) describing the aforementioned frequency of mentions in WHO Western Pacific Region’s Regional Committee Meetings (RCM) from 2010 to 2019; and 4) examining whether, and if so where and how, tobacco control and related key words (e.g. smoking) are discussed in the human rights treaty body materials for the Pacific Island Countries.

## Methods

We broadly adhere to a scoping review framework as outlined with Munn et al. (2018), noting that our literature comprises documents from UN bodies [28], as published research on this field is scarce. We focused on four potential sources of data. These were selected as a purposive sample of data based on the authors’ experience and the fact that this was the first known attempt to describe the (on the record) nexus of tobacco control and human rights within global governance forums.

We selected two global forums where States’ tobacco control and human rights priorities may normatively manifest: (1) WHA, held annually, representing one of the pinnacles for global health governance and norm-setting [29]; and (2) COPs for the WHO FCTC, where global tobacco control decision making takes place. To verify possible differences in the global from the regional debate we selected one WHO region, WHO Western Pacific Region (WPR), and (3) reviewed one regional forum, the annual WPR’s RCMs, to assess whether and how tobacco control and human rights are engaged in at a sub-global level. Our reason for selecting this region is described below. Finally, (4) we review human rights treaty body documents for a sample group of countries consistent with the regional review and thus select the Pacific Island Countries, which are members of the WPR [30]. Both authors developed the strategies for document review, but one of us (NRS) conducted the primary reviews, shared results with the other author (SAB), and jointly we discussed needs to refine the search, broaden the search words utilized and through an iterative process discussed inclusion or exclusion of mentions of tobacco in human rights in the results.

We started with a review of WHA resolutions speaking to tobacco since the WHO’s founding and cross-reviewed for references to human rights. We did this review through a word search of WHA resolutions using the words “tobacco” and “smoking”. This initial review was conducted to inform whether this was an exercise worth pursuing further, since we were not aware of a previous description of WHA resolutions linking tobacco and human rights.

We followed this overarching review with a detailed look at WHA documents from the past 10 years (2010 to 2019) – agenda, addresses, assembly reports, committee reports, and State reports/pronouncements – to identify references to “tobacco”, “smoking”, “human right”, and “right”. Other materials that were patently unrelated to tobacco control were not reviewed (e.g. budget reports). This period was selected as it is both recent and aligns with the rising prominence of the WHO FCTC and that of human rights in tobacco control. All materials reviewed were in English, and typically, as per WHO practice, are available in other UN languages. If a word search of the document identified a discussion on tobacco or human rights, the document was scrutinized in more detail to ensure that the context of the discussion, or germane discussions that may had escaped our initial search were found. We found that, overall, the word search of these documents accurately identified the discussions relevant to our search, minimizing the risk that we missed important data points.

We assessed WHO FCTC COP materials for references to human rights in tobacco control, following the same approach as with WHAs. COP began in 2004 with COP1. For our study period (2010–2019), session and committee reports as well as other available materials from COP4 (2010) to COP8 (2018) were reviewed for mentions of human rights.

As with WHA documents, we assessed WHO Western Pacific RCM materials – e.g. agenda, addresses, committee reports – for human rights and tobacco control mentions, from their annual meetings between 2010 and 2019.

We utilized the repository of documents from the human rights treaty bodies that is maintained by the UN Human Rights Council as a fourth source of data [9, 10]. States that have ratified a treaty regularly submit materials to that treaty’s overseeing committee, who in turn issue reports and recommendations to improve compliance; what States report, and committees ask about, can indicate whether tobacco control is seen as a fulfilment for a treaty’s principles if not also provisions. We accessed this repository in January 2020, and reviewed 1) State reports, 2) Compilations of UN information related to each State’s human rights commitments, 3) the Committee’s Concluding Observations (for UPR, the Working Group’s Report), 4) Lists of Issues identified by the respective Working Group or Committee to be addressed by the State, and 5) any available statements from the State party with respect to their submission. Initial keyword search terms included “tobacco” and “smoking” with standard search term snowballing; documents were dated 2010–2019. We limited our initial review to 15 Pacific Island Countries that are members of the WPR: Palau, New Zealand, Marshall Islands, Fiji, Kiribati, Federated States of Micronesia, Nauru, Papua New Guinea, Samoa, Solomon Islands, Timor-Leste, Tonga, Tuvalu, Vanuatu, and Philippines [30]. These countries within WPR were selected as a sample representing a range of social, political, economic and cultural contexts that may gauge whether human rights and tobacco control are integrated at that level of health governance.

NRS collected data and reviewed documents gathered from the noted sources; SAB provided guidance and contributed to analysis.

### Selecting WPR and Pacific Island Countries

The WPR was also selected purposively as (1) the Pacific Island Countries, which lay within this region, are understudied in tobacco control contexts, yet (2) the Western Pacific Regional Office (WPRO) of the WHO is highly engaged in tobacco control development and WHO FCTC implementation, with all 27 members having ratified the convention [31], and (3) regional governments, especially Pacific Island Countries which vary in their development stage and domestic resource capacities, have expressed their interest in legislative and governance strengthening for public health, particularly for children [6, 32–36]. Smoking prevalence across the islands varies significantly, as does availability of survey data, though data indicate that tobacco use prevalence is high overall for adults and youth in most Pacific Island Countries [37].

## Results

### WHA mentions for human rights and tobacco control

Three WHA resolutions were identified where “tobacco” and “right” were mentioned in the same context: WHA31.56 (1976), noting the “rights of non-smokers” [38]; WHA37.14 (1986), also noting the “rights of non-smokers” [39]; and WHA44.26 (1991), noting the “right to health” for nonsmokers [40]. These resolutions encouraged legislative and policy efforts to create environments free from smoke and smoking.

Table 1 shows mentions for human rights, tobacco control, and the WHO FCTC in WHA meetings from 2010 to 2019. Both tobacco control and human rights were mentioned in each WHA from 2010 to 2019, however the nature and depth of these mentions varies significantly. For the most part tobacco and human rights are not cited in the same context. WHA63 (2010) for instance, had multiple references to tobacco as a health concern and especially among populations like children but did not mention the CRC as a supporting rationale for public health measures to protect children’s health from tobacco use and exposure [41]. WHA64 (2011) made several references to the Universal Declaration of Human Rights, the right to health, and human rights treaties such as CEDAW, whereas tobacco is barely noted [42].

**Table 1:** World Health Assembly mentions for human rights, tobacco control, and WHO Framework Convention on Tobacco Control (2010–2019)

World Health Assembly	Human Rights Mentioned	Tobacco, Tobacco Use, Tobacco Control Mentioned	WHO FCTC Mentioned	Human Rights + Tobacco Control Mentioned	Observations
*Sixty-Third Assembly, 2010*	X	X	X		Human rights generally mentioned or in the context of other subject matters (sexual and gender rights, intellectual property rights)
*Sixty-Fourth Assembly, 2011*	X	X	X		Human rights and tobacco control minimally mentioned, though human rights treaties are referenced
*Sixty-Fifth Assembly, 2012*	X	X	X		Tobacco control mentioned significantly, but human rights minimally
*Sixty-Sixth Assembly, 2013*	X	X	X	X	One committee report - Fifth Report for Committee A - discusses tobacco control, WHO FCTC, and with limited mention human rights i.e. noting the right to the highest attainable level of physical and mental health
*Sixty-Seventh Assembly, 2014*	X	X	X	X	Human rights to health and tobacco mentioned in the context of controlling noncommunicable disease, primarily noting the right to the highest attainable level of physical and mental health
*Sixty-Eighth Assembly, 2015*	X	X	X		Human rights minimally mentioned; tobacco control minimally mentioned, primarily in the Sixth Report of Committee A
*Sixty-Ninth Assembly, 2016*	X	X	X	X	WHA called for synergies between WHO and WHO FCTC, noting the right to health as an embodiment in both the WHO and the Convention
*Seventieth Assembly, 2017*	X	X	X	X	WHA noted COP7 decision for international cooperation on issues of business, human rights, and tobacco control
*Seventy-First Assembly, 2018*	X	X	X	X	Second Report of Committee A notes the right to health as essential in controlling noncommunicable disease e.g. noting the right to the highest attainable level of physical and mental health, with mentions of tobacco and WHO FCTC
*Seventy-Second Assembly, 2019*	X	X	X	X	Notes the COP8 reference to continued importance for international cooperation regarding tobacco control, SDGs, and human rights

*Abbreviations*: *COP* Conference of the Parties, *SDG* Sustainable Development Goal, *WHA* World Health Assembly, *WHO* World Health Organization, *WHO FCTC* WHO Framework Convention on Tobacco Control

For tobacco, the role (and ratification) of the WHO FCTC becomes more frequently referenced in WHA materials from 2013 forward. WHA66 (2013) committed the WHO and member states to adhere to a human rights approach in the Action Plan for the Prevention and Control of Noncommunicable Diseases 2013–2020 and discussed tobacco 77 times with multiple calls to implement the WHO FCTC as part of this plan [43]. A committee report from WHA66 also discussed tobacco control and the WHO FCTC, with reference to the right to health as a framing for tobacco control. WHA67 (2014) continued this linkage between the right to health and tobacco control, suggesting that the right to the highest attainable level of physical and mental health provided a basis for enacting tobacco control measures and ratifying the WHO FCTC [44]. WHA69 recognized that the right to health was embodied in the WHO Constitution and the WHO FCTC [45].

### WHO FCTC COP mentions for human rights

COP meeting documents in 2010 (COP4), 2012 (COP5), 2014(COP6), 2016 (COP7), and 2018 (COP8) were reviewed (Table 2). COP7 introduced human rights and the SDG to the COP agenda and called for international cooperation with other UN human rights organs (e.g. OHCHR) to plan for a human rights-based approach to implementing the WHO FCTC and tobacco control [46]. COP8 revisited COP7’s call for greater cooperation with human rights organs in a status report [47]. Human rights and tobacco control within COP7 and COP8 focused on the role of business and government regulation thereupon, with particular emphasis on the tobacco industry.

**Table 2:** World Health Organization Framework Convention on Tobacco Control Conference of the Parties mentions for human rights (2010–2018)

WHO FCTC COP	Human Rights Mentioned	Human Rights + Tobacco Control Mentioned	Observations
*COP4, 2010*			No discernable mentions
*COP5, 2012*			No discernable mentions
*COP6, 2014*			No discernable mentions
*COP7, 2016*	X	X	A report from the Secretariat “*International cooperation for implementation of the WHO FCTC, including implementation of the 2030 Agenda for Sustainable Development, the global NCD targets and human rights*” noted the role of the WHO FCTC and tobacco control in the SDG, potential for collaboration with other UN agencies (UNICEF, Human Rights Council) to advance the discussion of tobacco control in the human rights context with particular regard to tobacco industry practices. The report further noted the role human rights play in implementing the WHO FCTC and proposed a framework for cooperation with other agencies and civil society and national governments that includes human rights organs.
*COP8, 2018*	X	X	Human rights mentioned with respect to COP7’s call for cooperation with human rights organs in the United Nations, but no new or significant discussion

*Abbreviations*: *WHO FCTC* WHO Framework Convention on Tobacco Control, *COP* Conference of the Parties, *NCD* Noncommunicable disease, *SDG* Sustainable Development Goals

### WHO Western Pacific RCM mentions for human rights and tobacco control

The WHO WPR’s RCMs from 2010 to 2019 more frequently linked tobacco control with human rights and human rights law than the WHAs (Table 3). RCM64 (2013) did not specifically address tobacco, however this meeting did include discussion on non-communicable disease and noted that non-communicable disease strategies must be “formulated and implemented in accordance with international human rights conventions and agreements” [33, 48]. RCM67 (2016) discussed the SDGs and within that context the role of the WHO FCTC and human rights treaties in reaching them [49].

**Table 3:** Western Pacific Regional Committee Meeting mentions for tobacco control, human rights, World Health Organization Framework Convention on Tobacco Control (2010–2019)

WPR Regional Committee Meeting	Human Rights Mentioned	Tobacco, Tobacco Use, Tobacco Control Mentioned	WHO FCTC Mentioned	Human Rights + Tobacco Control Mentioned	Observations
*Sixty-First Session, 2010*	X	X	X		Human rights often mentioned in the context of health systems and gender equity; tobacco control and WHO FCTC mentioned in context of health promotion
*Sixty-Second Session, 2011*	X	X	X		Mentions on tobacco industry interference in lawmaking and regulatory functioning
*Sixty-Third Session, 2012*	X	X	X		Mentions human rights treaties, however no connection between these treaties and tobacco-use disparities
*Sixty-Fourth Session, 2013*	X	X	X	X	Not specifically to tobacco, but “Western Pacific Regional Action Plan for the Prevention and Control of Noncommunicable Diseases (2014–2020)” states “Prevention and control strategies must be formulated and implemented in accordance with international human rights conventions and agreements.”
*Sixty-Fifth Session, 2014*	X	X	X	X	The Tobacco Free-Initiative Regional Action Plan (2015–2019) notes that the WHO FCTC recognizes the right to health; calls on members to identify civil society organizations concerned with women’s and children’s rights to work with on tobacco control; adds the inclusion of a rights indicator for evaluating country tobacco control measures; and calls on WHO and WPRO to provide technical assistance for countries and groups working to advance human rights and health as well as “advocate for the inclusion of tobacco control in the global, regional, and national health development agendas” including those pertaining to the SDG, CRC, and CEDAW.
*Sixty-Sixth Session, 2015*	X	X	X		Human rights and tobacco control were minimally mentioned in this RCM
*Sixty-Seventh Session, 2016*	X	X	X	X	Human rights and tobacco control are generally mentioned *de minimis*, however included substantial discussion on the SDG which included discussion on human rights, tobacco control, WHO FCTC, and human rights treaties e.g. CRC and CEDAW
*Sixty-Eighth Session, 2017*	X	X			RCM noted the CRC and a Human Rights Council resolution pertaining to the right to health for children with respect to food marketing, however this did not manifest in tobacco control
*Sixty-Ninth Session, 2018*	X	X	X		Human rights and tobacco control were minimally mentioned in this RCM
*Seventieth Session, 2019*		X	X		Tobacco control was a major agenda item for this RCM, however human rights were insignificantly mentioned

*Abbreviations*: *CEDAW* Convention on the Elimination of all forms of Discrimination Against Women, *CRC* Convention on the Rights of the Child, *RCM* Regional Committee Meeting, *WPRO* Western Pacific Regional Office, *WHO* World Health Organization, *WHO FCTC* WHO Framework Convention on Tobacco Control, *SDG* Sustainable Development Goals

WPRO’s Tobacco-Free Initiative has advocated for human rights as a facet to tobacco control, with both the 2010–2014 Action Plan [32], and 2015–2019 Action Plan introduced in RCM65 and adopted to by the WPR States [33], stating that the WHO FCTC “affirms the right of all people to the highest standard of health.” The Action Plans also directed WPRO (and WHO) to advocate for tobacco control’s inclusion into relevant agendas including the SDGs and before the pertinent committees overseeing human rights treaties (CRC, CEDAW). The 2015–2019 Action Plan also specified for nations and WPRO to identify and support advocacy groups working to advance women’s and children’s rights with the incorporation of tobacco control into that advocacy [33].

### Pacific Island Countries’ mentions of tobacco control at UN human rights treaty bodies

All member States of the United Nations submit UPR reports with respect to their general commitment to human rights, which are reviewed within the Human Rights Council [50]. Each State submits documents to the UPR Working Group (composed of representatives of other States) including a national report and background documents, and may respond to questions posed by the Working Group in their submissions. Civil society and third-party groups may likewise submit parallel reports identifying human rights issues or grievances to which the State under review should address. The Working Group issues its Report, following review, with recommendations for where and how a State may improve.

The process outlined above is the same for State reporting to human rights treaty bodies, with the change being that committees established under those respective treaties (e.g., Committee on the Elimination of All Forms of Discrimination Against Women, which oversees implementation of and compliance with CEDAW) review each State’s submission. The reporting mechanisms are mandatory for treaty members, though the regularity for reporting is not uniform.

Table 4 captures which reports and which countries mention tobacco control, smoking, and/or the WHO FCTC. Tobacco control was mentioned in 47 documents and under 6 human rights treaties. Most tobacco mentions (25 separate reports) occurred under the CRC (Table 4). UPR and CEDAW documents likewise made note of tobacco control efforts or tobacco-related disparities and prevalence, though infrequently. Mentions of tobacco included recognition of tobacco control laws enacted/proposed as well as noting particular disparities in vulnerable populations.

**Table 4:** Tobacco and WHO Framework Convention on Tobacco Control mentions before human rights treaty bodies for the selected countries (2010–2019)

Document Type	2010	2011	2012	2013	2014	2015	2016	2017	2018	2019
**Convention on the Rights of the Child**										
*State Report*			Tuvalu			Nauru	Marshall Islands; New Zealand; Palau	Federated States of Micronesia; Solomon Islands	Tonga	Philippines Tuvalu
*List of Issues and Questions from the Committee*				Tuvalu					Federated States of Micronesia; Palau; Tonga	Tuvalu
*Committee’s Concluding Observations*		Marshall Islands		Tuvalu	Fiji		Nauru; Samoa	Vanuatu	Palau; Solomon Islands	Tonga
**Convention on the Elimination of All Forms of Discrimination Against Women**										
*State Report*			Samoa			Kiribati	Fiji; Marshall Islands; Nauru; New Zealand			
*List of Issues and Questions from the Committee*										
*Committee’s Concluding Observations*			Samoa							
**Universal Periodic Review**										
*State Report*	Federated States of Micronesia	Nauru; Samoa; Solomon Islands				Federated States of Micronesia			Tonga; Tuvalu; Vanuatu	
*Working Group Report*			Philippines						Vanuatu	
*UPR Compilation of UN Information*	Kiribati									
**International Covenant on Economic, Social, and Cultural Rights**										
*State Report*								New Zealand		
*Committee’s Concluding Observations*			New Zealand							
**Convention on the Elimination of all forms of Racial Discrimination**										
*State Report*			New Zealand							
*List of Issues and Questions from the Committee*										
*Committee’s Concluding Observations*										
**Convention on the Rights of Persons with Disabilities**										
*State Report*										Palau; Tuvalu
*List of Issues and Questions from the Committee*										
*Committee’s Concluding Observations*										

A glossary of treaty body terminology is available online [51]

Table 5 shows how many documents mention tobacco per country in the sample countries. We did not find any tobacco mentions within the reviewed materials for Papua New Guinea or Timor-Leste. Ratification for the WHO FCTC is referenced in Nauru’s report (2016) and Palau’s report (2016) to the CRC in 2016 [52, 53]; and the CRC Committee’s observations to Fiji (2014) and New Zealand (2011) [54, 55].

**Table 5:** Number of documents mentioning tobacco or smoking in human rights treaty body documents from 2010 to 2019 by Pacific Island Countries, in alphabetical order

Pacific Island Country	# of UN Human Rights Treaty Body documents mentioning tobacco or smoking
Fiji	2
Federated States of Micronesia	4
Kiribati	2
Marshall Islands	3
Nauru	4
New Zealand	5
Palau	4
Philippines	2
Samoa	4
Solomon Islands	3
Tonga	4
Tuvalu	7
Vanuatu	3

#### Example of Pacific Island Countries key points linking tobacco to human rights among select human rights treaty body documents

In most instances mentions for tobacco or smoking were passing, such as a statement that reducing tobacco use is a priority or that a country passed a tobacco control law without further elaboration.

#### Federated States of Micronesia

Micronesia mentioned tobacco and smoking multiple times in its state party report to CRC in 2017 [56], speaking to the commitment from its maternal and child health programs to reducing tobacco use and access for children and pregnant women. The CRC Committee was likewise interested in reviewing tobacco abuse among children in its List of Issues to review for the country [57]. Tobacco and smoking were also mentioned in Micronesia’s Universal Periodic Review reports to the Human Rights Council in 2010 and 2015 [58, 59].

#### Marshall Islands

In 2018, the Committee on the Rights of the Child recommended to the Marshall Islands they develop policies and laws to better address smoking among youth. This observation came after the Marshall Islands’ submitted its 2016 country report wherein it noted the need to improve reductions in adolescent and youth tobacco use and pointed to several of its laws that purported to regulate tobacco and betel sale, production, and distribution [60].

#### Nauru

In 2016, the Committee on the Rights of the Child (which oversees the CRC) recommended that Nauru “immediately address tobacco and alcohol use by children”, suggesting that Nauru was not adequately informing youth as to the risks for personal harm or had an education system that instructed suitable “life skills … on preventing such abuse.” [61] The committee noted that this recommendation stems from General Comment 4 (2003) [62], which both reiterated the right to health and other rights for children and connected CRC to ICESCR and General Comment 14 illuminating the right to health in that treaty [12].

#### New Zealand

New Zealand’s materials mention tobacco and smoking primarily by reference to successes in its public health programs to reduce consumption among some populations. Nevertheless, the Committee for Economic, Social, and Cultural Rights expressed its concern regarding tobacco use and smoking among the Maori and Pasifika populations in 2012 [63]. The Committee’s concerns were reasonable, particularly as shown by Ball et al. that smoking and tobacco use remains prevalent among these populations, including among youth [64].

#### Palau

Palau has received praise for addressing tobacco as a human rights concern before the Human Rights Council [65]. In 2018 the Committee on the Rights of the Child expressed its concerns over child exploitation by the tobacco industry as well as high tobacco consumption among youth [66]. Palau, to its credit, recognized these concerns as well in its submission to the CRC in 2017 [53].

## Discussion

In the 10 year period studied, 2010–2015 saw few instances of human rights entering official promulgations in WHA and COP; from 2016 forward human rights and tobacco control became more frequently mentioned within each other’s orbits. This may be no coincidence; COP7 in 2016 connected tobacco and human rights [46]. It is possible that advocates are influencing national governments and/or individuals within them with respect to framing tobacco control in human rights terms [14, 15, 26, 67–70]. Still, these conversations are not uniformly occurring at all levels of tobacco control and public health, globally as well as within the Pacific Island Countries and WPR.

The paucity of these conversations is concerning, as the relationship between health and human rights has been well established and applied in other settings such as HIV/AIDS [71]. The body of literature is expansive with respect to health and human rights, and States do discuss or agree to other health subjects in human rights terms e.g. mental health [72], and broadly women’s and adolescent health [73], but not on tobacco. Though advocates have built on this foundation and made connections between tobacco control and human rights, their advocacy appears to not have moved States to frame tobacco control in human rights terms significantly or substantively.

### Global and WPR health governance have seldom broached human rights and tobacco control in the same context

The majority of WHA and WPR’s RCM documents that discuss tobacco control do so separately from a human rights context; likewise, human rights references are generalized citations to WHO’s Constitution or specified to another human right subject matter such as sexual and reproductive health. The nexus between human rights and tobacco control is, however, further explored outside of WHA and WPR’s RCM events, with the RCMs identifying human rights connections in their work on tobacco control. Such efforts, though, might represent the interests of the WHO and WPRO more than the States party in seeing tobacco control and human rights joined, as perhaps indicated by the WPRO’s strategic plans.

WPR is one of several WHO regions, and the Pacific Island Countries a subset of the concert of nations. We selected this region for diversity in national development levels and future studies should include assessments for human rights and tobacco control linkages in the other WHO regional bodies, as well as a comprehensive country-by-country review of tobacco control mentions by the human rights treaty bodies.

Several Pacific Island Countries signed the Denaru Declaration on Human Rights and Good Governance in 2015, which noted non-communicable disease (including tobacco use as a driver of these diseases) as an emerging human rights issue in their region that required new legislation, policy, and practices to address [74]. How this commitment is being operationalized in policies is yet to be determined.

### Human rights treaty bodies, and especially the CRC, are receptive to tobacco control as a dimension of a State’s human rights obligations

Human rights treaty bodies’ mentions for tobacco control and tobacco use were rarely substantive discussions, and often statements on a country passing a law without attributing that law to a human rights obligation per se. Still, that a country mentioned that a tobacco control measure was passed in support of, inter alia, a child’s right to health, is noteworthy. Committee questions and concluding observations noting tobacco and smoking is likewise an important development towards potentially conceptualizing tobacco control as a component (however sized) to treaty compliance. The CRC may be especially compatible for tobacco control argumentation, and presents a mechanism that advocates may work through to pressure governments to pass policies restricting access to and use of tobacco products for youth. Similarly, with the CRC’s apparent interest, advocates may consider other treaty bodies (e.g. CEDAW, ICESCR) as amenable to tobacco control and WHO FCTC compliance as a part of their treaty’s expectations as well.

### WHO FCTC as a bridge between human rights law, tobacco control, and development?

COPs have begun to explore human rights through tobacco control. COP7 provided an exploratory basis for the WHO FCTC to officially partner with human rights organizations and jointly pursue human rights and tobacco control [75]. The decision emerging out of this discussion – FCTC/COP7(26) – linked the tobacco industry and human rights concerns to the UN Human Rights Council’s resolution on human rights in business (A/HRC/RES/26/9) and further invited the WHO FCTC Secretariat to “collaborate with existing United Nations mechanisms and processes working on issues of business and human rights” and to use a “human rights framework and development to tackle the global tobacco epidemic” that the tobacco industry capitalizes upon [75]. The drive behind connecting the tobacco industry to human rights’ violations included the industry’s efforts to scale up consumption for tobacco products “particularly in low-income countries,” with the WHO FCTC providing a basis to collaborate with other UN agencies to “protect public health from the commercial and vested interests of the tobacco industry” [75]. COP8 (2018) did not go further than COP7 on human rights though, with the agenda and discussion limited to updates on FCTC/COP7(26) [47].

The SDG, which base their principles in human rights [7], envision ratification of the WHO FCTC as essential to meeting its health goals, and meeting the SDG has spurred many WPR countries to develop and improve their laws and regulations including for tobacco control [6]. Whether the SDG see the WHO FCTC as also a human rights tool is arguable [76], though the WHO FCTC’s proponents see the treaty as a potential newcomer to human rights legal canon [2, 19, 23]. Country priorities in development may be influenced through human rights advocacy for WHO FCTC ratification, implementation, and strengthening, and normative framings of tobacco control as related to human rights proffered through organizations like WHO and WPRO may support tobacco control advocacy within national development plans.

### Limitations

Our study includes several limitations. We utilized search terms that were generally standard and frequent in any discussion on tobacco, tobacco control, smoking, and human rights; we reviewed only the materials made available by the organizational meetings we looked at (WHA, COP, RCM, treaty body meetings), which means materials not accessible or materials from other gatherings were not reviewed. We did not review materials that were patently off-topic for tobacco control – e.g. an annual budget report. Our study limitation to WPR and the Pacific Island Countries impacts the scope of our findings as human rights and tobacco control mentions in other regions might be more frequent. We did not review national policies to determine if any tobacco control legislation or policy have operationalized human rights in its implementation.

### Implications for future research

As a first attempt to identify the nexus of tobacco control and human right in these global forums, we provide a roadmap for subsequent analyses which will indicate if the Pacific Island Countries and WPR’s RCM are reflective of the global norm or an outlier when it comes to human rights and tobacco control as an integrated concept for advocates and policymakers. If more countries report their tobacco control efforts through the lens of their human rights commitments, the likelihood of tobacco control becoming a greater component to human rights increases. Direct linkages between WHO FCTC articles, SDG priorities, and human rights obligations under pertinent treaties may prove to be a compelling argument for local, regional, and global tobacco control, should advocates and authorities find themselves willing and able to engage at this intersection of health and human rights. Understanding if and how countries are moving from stated commitments to incorporating human rights in tobacco control program implementation would contribute to the realization of a rights-based approach to tobacco control.

## Conclusion

While some discussion is occurring at the intersection of human rights and tobacco control, this conversation remains in its infancy. Human rights remain outside the framing for most delegates to WHA, COP, and WPR’s RCMs. The discussion of tobacco control within those global and regional health governance bodies relates strongly to traditional conceptualizations around public health and development, the latter becoming more important with the SDG. Where they have occurred, connections to human rights are generalized, e.g. framing of the right to the highest attainable standard of physical and mental health as informing all that a country should do to promote overall health, as compared to a specific scrutiny of a country’s human rights obligations and how tobacco control supports or fulfills them. Tobacco and smoking are uncommon mentions in human rights treaty proceedings as well, with the preponderance of mentions occurring under the CRC. This may be strategic, if narrowing the scope of a human rights-based argument for tobacco control, as children do enjoy special protections and social distinction within many societies [68, 70, 77–79]. Human rights advocates, and observers, may be moving closer towards tobacco control [19, 25, 80]. Human rights treaty bodies are, however, deferential to a State’s ratification of the WHO FCTC and enacting legislation to fulfill WHO FCTC commitments.

## Data Availability

Not applicable.

## Abbreviations

COP: Conference of the Parties
CEDAW: Convention on the Elimination of all forms of Discrimination Against Women
CRC: Convention on the Rights of the Child
HR: Human Rights
ICESCR: International Covenant on Economic, Social, and Cultural Rights
RCM: Regional Committee Meeting
SDG: Sustainable Development Goals
UN: United Nations
UPR: Universal Periodic Review
WPR: Western Pacific Region
WPRO: Western Pacific Regional Office
WHO FCTC: WHO Framework Convention on Tobacco Control
WHA: World Health Assembly
WHO: World Health Organization
